# Parliamentary reaction to the announcement and implementation of the UK Soft Drinks Industry Levy: applied thematic analysis of 2016–2020 parliamentary debates

**DOI:** 10.1017/S1368980024000247

**Published:** 2024-01-24

**Authors:** Catrin P Jones, Emma R Lawlor, Hannah Forde, Dolly RZ van Tulleken, Steven Cummins, Jean Adams, Richard Smith, Mike Rayner, Harry Rutter, Tarra L Penney, Olivia Alliot, Sofie Armitage, Martin White

**Affiliations:** 1 MRC Epidemiology Unit, University of Cambridge, Cambridge, CB2 0QQ, UK; 2 Population Health Innovation Lab, Department of Public Health, Environments & Society, Faculty of Public Health and Policy, London School of Hygiene & Tropical Medicine, London, UK; 3 Faculty of Health and Life Sciences, University of Exeter, Exeter, UK; 4 Nuffield Department of Population Health, University of Oxford, Oxford, UK; 5 Department of Social and Policy Sciences, University of Bath, Bath, UK; 6 Global Food System and Policy Research, School of Global Health, Faculty of Health, York University, Toronto, Canada

**Keywords:** Government, Parliament, Political debate, Sugar tax, Soft Drinks Industry Levy, Health policy

## Abstract

**Objective:**

The UK Soft Drinks Industry Levy (SDIL) (announced in March 2016; implemented in April 2018) aims to incentivise reformulation of soft drinks to reduce added sugar levels. The SDIL has been applauded as a policy success, and it has survived calls from parliamentarians for it to be repealed. We aimed to explore parliamentary reaction to the SDIL following its announcement until two years post-implementation in order to understand how health policy can become established and resilient to opposition.

**Design:**

Searches of Hansard for parliamentary debate transcripts that discussed the SDIL retrieved 186 transcripts, with 160 included after screening. Five stages of Applied Thematic Analysis were conducted: familiarisation and creation of initial codebooks; independent second coding; codebook finalisation through team consensus; final coding of the dataset to the complete codebook; and theme finalisation through team consensus.

**Setting:**

The United Kingdom Parliament

**Participants:**

N/A

**Results:**

Between the announcement (16/03/2016) – royal assent (26/04/2017), two themes were identified *1: SDIL welcomed cross-party 2: SDIL a good start but not enough.* Between royal assent – implementation (5/04/2018), one theme was identified *3: The SDIL worked – what next?* The final theme identified from implementation until 16/03/2020 was *4: Moving on from the SDIL*.

**Conclusions:**

After the announcement, the SDIL had cross-party support and was recognised to have encouraged reformulation prior to implementation. Lessons for governments indicate that the combination of cross-party support and a policy’s documented success in achieving its aim can help cement the resilience of it to opposition and threats of repeal.

Sugar sweetened beverages (SSB) are a major source of dietary sugar, with consumption associated with type 2 diabetes, dental caries and cardiovascular disease^(1)^. The WHO has supported efforts that target SSB to reduce sugar consumption globally and has outlined a range of policies and recommendations to achieve this^(2)^. Successful implementation of SSB taxes is therefore of significant international interest to policymakers and researchers^(3)^. Taxes and levies on SSB have been introduced in more than 50 countries globally in an effort to improve population diets and tackle diet-related ill health^(4)^.

In the United Kingdom (UK), the SSB tax scheme called the Soft Drinks Industry Levy (SDIL) came into force on the 6th April 2018^(5)^. Announced by the Chancellor of the Exchequer at the time, George Osborne, in his spring budget statement on the 16th March 2016, the SDIL aims to encourage manufacturers of SSB to reduce the sugar content of their products through reformulation^(6)^. Unlike most SSB taxes implemented elsewhere, for example Mexico^(7)^, Philadelphia, United States of America (USA)^(8)^, Barbados^(9)^ and Chile^(10)^, the SDIL is not primarily designed to influence consumer purchasing decisions by directly raising the price of drinks. Instead, the SDIL targets manufacturers by introducing a two-tier levy which varies depending on the level of added sugar; £0.24 per litre for drinks containing ≥8 g; £0.18 per litre for drinks containing ≥5 g and <8 g; and zero tax for drinks containing less than 5 g of total sugar per 100 ml^(5)^. The SDIL was purposefully announced 2 years before implementation to allow time for companies to prepare for and implement reformulation^(11)^.

Introducing a tax is controversial and often subject to opposition. Internationally, a number of SSB taxes have been repealed or threatened with repeal; Denmark repealed a soft drink tax in place since 1930 in January 2014^(12)^, Cook County, Illinois, USA, repealed a soda tax in place from 2nd August 2017–1st December 2017^(13)^, and in Philadelphia, USA, a soda tax was challenged in court, due to perceived disproportionate impact on deprived groups^(14)^, but survived^(15)^. Other public health policies have been subject to significant challenge, e.g. a New York portion size cap introduced on 2012 was repealed following a court challenge^(16)^, and the proposed UK ban on TV and online advertising of unhealthy foods is delayed until 2025^(17)^.

While there is good evidence that the SDIL achieved its primary aims (reformulation and reducing sugar consumption)^(18,19)^, like those policies above there have been threats to repeal it. In July 2019, Boris Johnson, in his successful campaign to become Prime Minister, suggested a review of the SDIL with the threat to repeal due to the perception that it is a ‘sin tax’ and ‘clobbers those who can least afford it’^(20)^. In September 2022, Prime Minister Liz Truss threatened to reverse the SDIL and other public health policies she deemed ‘nanny statist’^(21)^. In both cases, these threats were reconsidered, potentially due to a backlash from Members of Parliament (MP), policy advocates and academics^(22,23)^ These threats demonstrate that evidence of a policy’s ‘success’ does not necessarily protect it from repeal. However, the SDIL has survived repeal threats unlike many other SSB taxes internationally. Important learning for public health and policymakers can be found by examining the SDIL and its surrounding political context, to understand why it may have survived when similar policies elsewhere have not.

Parliamentary debate provides a window into the perception of, and reaction to, a policy by politicians. Parliament is the legislative body of the UK and contains two chambers: the House of Lords and the House of Commons. Debate in these houses provides a public platform for discussion between MP or peers, including government ministers. The purpose of parliamentary debate is primarily to test ideas and to place them on the political agenda, either as new proposals or surrounding existing legislation and to discuss, amend and approve legislation^(24)^. Through locally elected MP, the public and organisations are able to raise issues^(24)^ and debate provides a platform to ask questions of the sitting government. Although there are critics of the true power of the UK parliament, it has been suggested that parliamentary debate and questions can provide insights into reactions to new policy^(25)^.

Exploring parliamentary debate following the announcement of the UK SDIL provides an opportunity to explore not only the immediate parliamentary reaction to the policy but also the changing perceptions of the policy over time. The aim of this qualitative study was to explore reactions to the UK SDIL by examining parliamentary debate following its announcement in 2016 until 2 years following its implementation (16/03/2016–16/03/2020).

## Methods

To examine UK parliamentary debate between the announcement and implementation of the SDIL, we took an exploratory descriptive approach using Applied Thematic Analysis^(26)^ and qualitative and quantitative analysis techniques. Applied Thematic Analysis goes beyond quantitative content analysis by providing a more accountable and testable method than traditional methods of policy analysis^(27)^.

### Methods

Applied Thematic Analysis uses five steps: (1) planning and preparation, (2) data gathering and eligibility screening, (3) first-level analysis (familiarisation and mapping preliminary themes and codes), (4) second-level analysis (coding and codebook refinement), and (5) third-level analysis (interpreting data).

### Step 1: planning and preparation

An audit trail and analysis plan document were created in step one (available from the authors on request). Analyses were initially conducted in two time periods: announcement – implementation (16/03/2016–05/04/2018) and post-implementation (06/04/2018–16/03/2020). The 16th March 2020 was chosen as the end date for data collection as it is four years following the announcement of the SDIL. Analysis commenced in March 2020 and due to the size of the data corpus took considerable time. The inclusion of repeated data collection and analysis following this date was too resource-intensive, due to ATA and searches being conducted manually.

### Step 2: data gathering and eligibility screening

Hansard, a website containing transcripts of all UK parliamentary debate records^(28)^, was searched for documents discussing the SDIL in the House of Commons or House of Lords using the following search terms: ‘soft drinks industry levy’, ‘sugar tax’, ‘sugar levy’, ‘sugary drinks tax’, ‘soft drinks tax’, ‘soft drink tax’, ‘soft drink levy’, ‘fizzy drinks tax’ and ‘fizzy drink tax’. Transcripts were downloaded and then imported into NVivo 12^(29)^. CPJ conducted familiarisation and developed initial eligibility criteria. These were piloted on 10% of documents by CPJ and ERL until agreement was reached. Remaining documents were screened according to the final eligibility criteria, reference to the SDIL (directly by name or indirectly – e.g. referred to as ‘the sugar tax’) in any context. Documents that did not refer to the SDIL were excluded as were those that referred to sugar outside the context of diet, were personal reflections on the speaker’s own dietary behaviour or made reference to the SDIL. The final dataset consisted of 160 eligible documents, 90 from announcement – implementation and 70 post-implementation, 26 retrieved documents were excluded.

### Step 3: first-level analysis

CPJ first analysed three main debate documents regarding the SDIL (*Financial Statement 2016-03-16, Soft Drinks Industry Levy_ Funding for Sport in Schools 2017-01-10, Draft Soft Drinks Industry Levy (Enforcement) regulations 2018-02-07*) in full, selected as they were debates specifically on the SDIL and likely to be rich in relevant information. CPJ explored the data through structural coding by sentiment (for, against, neutral), political party, use of evidence and important actors, with the intention of conducting structural analysis on the full dataset. However, it became apparent that important nuances were missed, and thematic coding was pursued instead. All 160 documents were explored thematically, and codes generated from the three main documents were carried through to other documents, leading to the inclusion of all documents in our analysis.

To locate relevant SDIL text within non-SDIL specific debates, keyword searches for ‘sugar’ ‘levy’ ‘tax’ ‘fizzy’ and ‘drinks’ were conducted. A preliminary thematic codebook for the announcement-implementation period was created by CPJ following familiarisation, text tagging and recording of initial impressions. CPJ then coded each document by locating references to the SDIL along with any relevant surrounding text and refined the codebook accordingly.

### Step 4: second-level analysis

Second-level analysis involved systematic coding to build the codebook. CPJ coded all documents in the announcement–implementation period, while ERL and HF independently coded 10% (*n* 9) of documents containing the highest number of CPJ coding references. ERL and HF suggested alterations to existing codes or the inclusion of new ones, where appropriate. CPJ, ERL and HF then discussed and refined the codebook. This process was repeated, adapting the codebook from the pre-implementation period for the post-implementation period.

During analysis, the number of references to the SDIL following ‘royal assent’ of the policy (when a policy proposal becomes law by being granted ‘Royal Assent’ by the monarch) decreased. Additionally, a transition in the content of SDIL debate led to the addition of a third time period, prior to step 5 of analysis: announcement – royal assent (16//03/2016–26/04/2017), royal assent – implementation (27/04/2017–05/04/2018), post-implementation (06/04/2018–16/03/2020). A final combined codebook based on these reflections was produced by CPJ with agreement from all coders and applied to the full dataset.

### Step 5: third-level analysis

Third-level analysis involved generating quantitative summaries of the following: the distribution of included documents over time, whether documents and codes were from debates in the House of Lords or Commons, and the distribution of codes in the codebook over time. Distribution of these codes was explored graphically through the generation of histograms, no changes to findings resulted from these; therefore, frequencies only were used in final thematic generation. The aim of this final analytic step was to understand the frequency of each code to ensure they were emphasised accordingly during the generation of themes. Themes were then developed based on these frequencies and the codebook, with the frequency of codes within each time period used to prioritise codes for integration within themes.

## Results

**Fig. 1:**
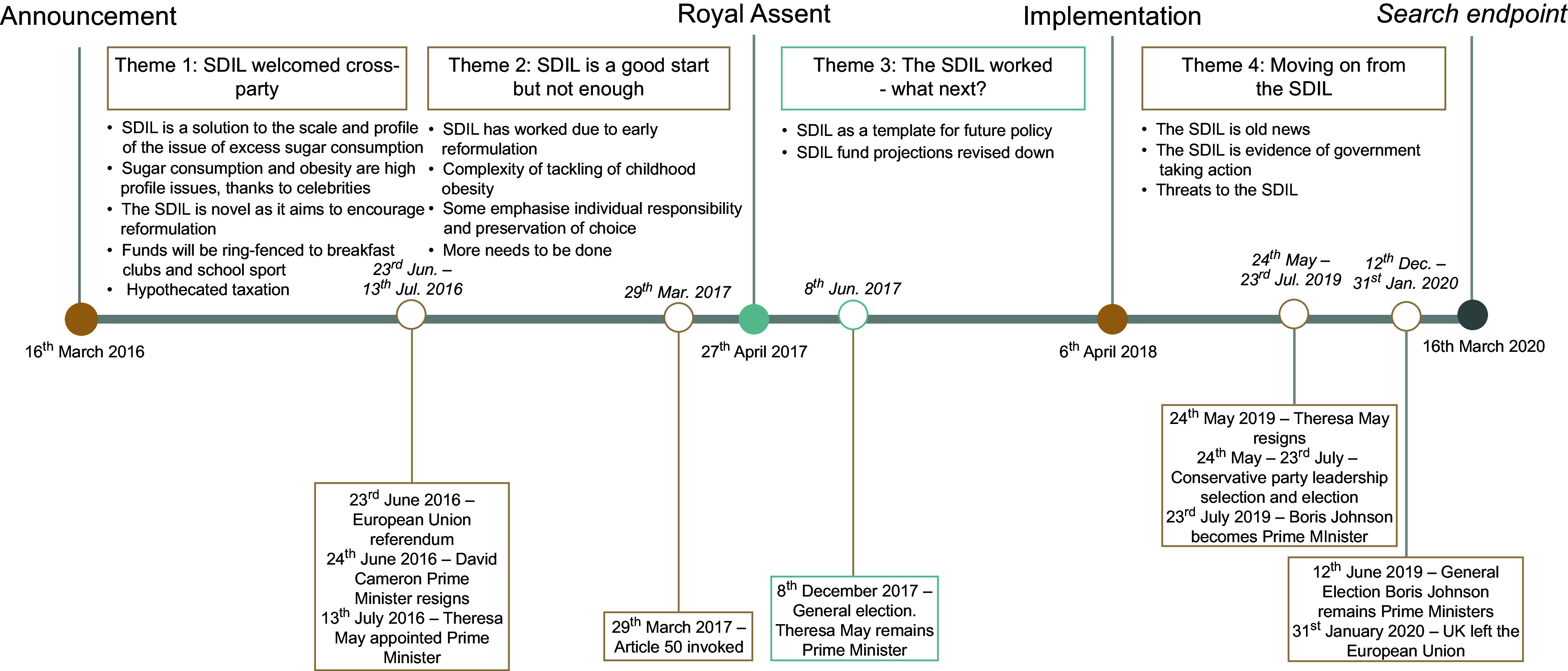
Results of an Applied Thematic Analysis of parliamentary reaction to UK Soft Drinks Industry Levy including external UK political events that occurred (16/03/2016 – 16/03/2020)

Four main themes spanned the time periods. Figure1 summarises these alongside external political events that occurred during this time period (Box 1).

Box 1 A description of key political events in the United Kingdom surrounding the announcement of the UK soft drinks industry levy until two years following implementation (16/03/2016 – 16/03/2020)

**Party elections and Leadership**

- On 07/05/2015, a general election was held, the Conservative party won a majority of seats in the House of Commons and David Cameron remained Prime Minister.
- David Cameron announced his intention to resign on 24/06/2016 and officially resigned as Prime Minister on 13/07/2016.
- Theresa May was appointed as Prime Minister on 13/07/2016, following a Conservative Party leadership process.
- An early general election was held on 08/06/2017 resulting in a hung Parliament. Following an agreement between the Conservative Party and the Democratic Unionist Party in Northern Ireland, Theresa May was able to form a minority government and remained Prime Minister.
- On 24/05/2019, Theresa May announced her intention to resign as Prime Minister.
- Between 24th May–23rd July, a Conservative Party leadership process led to Boris Johnson being appointed Prime Minister on 23/07/2019.
- A general election was held on 12/12/2019, and the Conservative Party won a majority in the House of Commons.

**Brexit**

- A referendum on whether the UK should remain a member of the European Union was held on 23/06/2016 resulting in the majority of votes cast to leave the EU.
- Article 50 of the Treaty of the European Union was invoked on 29/03/2017, which started the process of the UK leaving the EU with a formal date of 29/03/2019 when the UK would exit.
- This deadline was extended twice, firstly until 12/04/2019 and secondly 31/10/2019.
- The UK officially left the EU on 31st January 2020 with a ‘transition period’ until 31/12/2020.

Themes including quotes are presented below alongside the MP’s or peer’s name, their political party and constituency (where relevant), whether they are a member of government and their ministerial role, and the document name listed by Hansard.

### Announcement to royal assent: March 2016–April 2017

#### Theme 1: SDIL welcomed cross-party

The SDIL was welcomed by most MP and peers quoted in the included documents, with notable cross-party support. Some were surprised at the introduction of the policy, and the ‘U-turn’ the government appeared to take after previously stating it would not introduce an SSB tax.

*‘Colleagues on both sides of the House have talked about their support for the soft drinks levy and positive changes relating to childhood obesity’.*

*Kirsty Blackman (Scottish National Party) (Aberdeen North)*

*Finance (No.2) Bill 2017-04-18*

*‘I am grateful for the Government’s U-turn from their position before the debate in November, when they stated that they had “no plans to introduce a tax on sugar-sweetened beverages.”’*

*John McNally (Scottish National Party) (Falkirk)*

*Budget Resolutions and Economic Situation 2016-03-22*

##### SDIL is a solution to the scale and profile of the issue of excess sugar consumption

The scale of the problems associated with excess sugar consumption was strongly emphasised by MP, in particular associations with obesity, diabetes, dental health and the subsequent cost of these to the NHS, which contributed to the positive reception the SDIL received as it was seen as part of the solution to these problems.

*‘I also welcome the Government’s soft drinks levy. Such legislation is an important step in tackling obesity and the unhealthy diets that contribute to it. It has been found that one in three children between the ages of two and 15 is obese, and that 20% of the NHS budget is spent on dealing with health problems that are a direct result of unhealthy lifestyles’.*

*Baron Diljit Rana (Conservative Party) (Malone)*

*Queen’s Speech 2016-05-25*

##### Sugar consumption and obesity are high-profile issues, thanks to celebrities

Sugar consumption and obesity in children were discussed as high-profile issues, and in particular the role of celebrities and notable people or organisations in raising awareness. These issues were also linked to the SDIL being welcomed.

*‘I want to take this opportunity to pay particular tribute to Jamie Oliver and the Obesity Health Alliance, who have campaigned tirelessly on this issue and on the need for a joined-up Government obesity strategy’.*

*Peter Dowd, Shadow Financial Secretary to the Treasury (Labour Party) (Bootle)*

*Finance (No. 2) Bill 2017-04-25*

However, the high-profile nature of the public health issue due to celebrity involvement was also used as a critique of the SDIL. This included that it was announced only due to its status. The role of celebrity chef Jamie Oliver was particularly noted.

*Does the honourable gentleman agree that this tax, which has many ambiguities, simply indulges our celebrity chefs and gives them more credence than they deserve?*

*Mary Glindon (Labour Party) (North Tyneside)*

*‘… The sugar tax is a passion of TV chef Mr Jamie Oliver, who is just the latest in a line of celebrities…to use their position to influence public policy’.*

*Richard Fuller (Conservative Party) (Bedford)*

*Budget Resolutions and Economic Situation 2016-03-22*

##### The SDIL is novel as it aims to encourage reformulation

The cross-party support was further attributed to the perception of the SDIL as a novel and ground-breaking policy. The design of the SDIL and focus on encouraging manufacturers to reformulate was part of the perception that it was novel.

*‘I welcome the contribution that it will make as part of a wider strategy to tackle childhood obesity. It will encourage manufacturers to reformulate their products to bring in lower levels of sugar’.*

*Sarah Wollaston, Chair of Health Select Committee (Conservative Party) (Totnes)*

*The Economy and Work 2016-05-26*

##### Funds will be ring-fenced to breakfast clubs and school sport

A positive reception to the ring-fencing of funds to school sport and breakfast clubs was a critical part of the SDIL having cross-party support. For example, MP were supportive of role of the SDIL in increasing Treasury funds, which could be used to mitigate childhood obesity by paying for children’s breakfast clubs and sport.

*‘I welcome the announcement in the Budget of the sugar tax, and also the fact that the money raised will be spent on school sports’.*

*Liz McInnes, Shadow Minister for Communities and Local Government (Labour Party) (Heywood & Middleton)*

*Business of the House 2016-03-24*

The SDIL was perceived to be a ‘win-win’ policy: if the levy worked to encourage reformulation, it would improve the countries’ health via reduced sugar consumption, and if it did not encourage reformulation at least, it would generate funds to improve health via other mechanisms.

*‘I want to touch briefly on the sugar tax… Instinctively, I too am a low-tax Conservative and therefore cautious about this measure, but I warmly welcome the direction that this money will go in…I believe that the additional funding for sport in primary and secondary schools will be warmly welcomed’.*

*Michael Tomlinson (Conservative Party) (Mid Dorset & North Poole)*

*Budget Resolutions and Economic Situation 2016-03-17*

##### Hypothecated taxation

There was some scepticism about this type of levy and hypothecated taxation generally. In particular, speakers questioned how levy funds could be accurately predicted and what would happen if there was a shortfall in comparison to funds promised for school sports and breakfast clubs.

*‘I am worried because, on my calculation, reformulation, portion size, illicit sales and such things as cross-border shopping will mean that the figure raised will be more like £200 million to £300 million. That is a considerable shortfall on the amount quoted in the Budget last year. We must ask questions about hypothecated taxes and direct taxes’.*

*Will Quince (Conservative Party) (Colchester)*

*Soft Drinks Industry Levy: Funding for Sport in Schools 2017-01-10*

The government promised to fill any shortfall of funds accrued by the SDIL, when initial forecasts were revised down following the early reformulation conducted by industry between the SDIL announcement and implementation.

*‘Producers are already reformulating sugar out of their drinks, which means a lower revenue forecast for this tax…I can confirm that we will nonetheless fund the Department for Education with the full £1 billion that we originally expected from the levy this Parliament, to invest in school sports and healthy living programmes’.*

*Philip Hammond, The Chancellor of the Exchequer (Conservative Party) (Runnymede & Weybridge)*

*Financial Statement 2017-03-08*

#### Theme 2: SDIL a good start but not enough

##### SDIL has worked due to early reformulation

The SDIL was described as a success due to the early reformulation undertaken by industry prior to implementation. Discussion often centred around early reformulation and policymakers discussed the SDIL in a way that suggested they had concluded it had worked even prior to implementation.

*‘It has been a mark of the success of the progress made with this policy that reformulation is already taking place, and it is therefore expected that in fact £1 billion will not be raised’.*

*Jane Ellison, The Financial Secretary to the Treasury (Conservative Party) (Battersea)*

*Finance (No.2) Bill 2017-04-18*

*‘I am even more pleased that the levy is already working, with Tesco – once my employer, so that is good to hear – and the manufacturers of Lucozade, Ribena and Irn-Bru among those already committing to reformulate their drinks and reduce added sugar’.*

*Baroness Neville-Rolfe (Conservative Party) (Chilmark)*

*Finance (No.2) Bill 2017-04-26*

This early reformulation linked to discussion that the SDIL could be a template for future policy.

*‘I hope that the Chancellor will consider not only the sugar tax, but a levy on tobacco companies…then ensuring that all the money raised goes directly to funding local health initiatives’.*

*Bob Blackman (Conservative Party) (Harrow East)*

*Defending Public Services 2016-05-23*

##### Complexity of tackling childhood obesity

‘Complexity’ arguments surrounding the food system and obesity were discussed from all sides and were repeated often, specifically that obesity and food system problems are complex with no single solution. The phrase ‘there is no silver bullet’ was used often and in the context of speakers both for and against the SDIL.

*‘I do agree with the sugar tax, but it is not a silver bullet. To deal with child obesity, there needs to be long-term, careful planning, and there needs to be a change in lifestyles as well’.*

*Albert Owen (Labour Party) (Ynys Môn)*

*Budget Resolutions and Economic Situation 2016-03-17*

*‘The levy is a bold and brave move, but it is only a small part of the efforts we need to make to tackle this problem. Unless we tackle it from a multitude of directions with a number of different strategies, we will not make progress. There is no one silver bullet’.*

James Davies (Conservative Party) (Vale of Clwyd)

Finance (No.2) Bill 2017-04-18

Those against the SDIL also used complexity arguments. They discussed that targeting a single product alone will not impact health and that changing consumer behaviour is also required and won’t be achieved by the SDIL.

*‘Let us just say that I am sceptical about a levy on sugar. It is one of those policies that sounds good and catches the headline, but it has no sound evidential base’.*

*Ian Paisley (Democratic Unionist Party) (North Antrim)*

*Defending Public Services 2016-05-23*

##### Individual responsibility and preservation of personal choice

Some speakers had reservations about the SDIL and expressed views using arguments based on individual responsibility. They argued for the importance of individual responsibility for health and discussed that the government should not take away people’s choice. They stated that individuals are responsible for their consumption and argued that exercise and education should be promoted rather than taxes levied.

*‘I believe we should cut people’s taxes and then let them make their own choices… These companies, of course, will find a way around it – they will probably just ensure that a Diet Coke costs the same as a normal bottle of Pepsi’.*

*Edward Leigh (Conservative Party) (Gainsborough)*

*Budget Resolutions and Economic Situation 2016-03-17*

*‘However, parents must bear much of the responsibility for ensuring that children lead a healthy and active lifestyle…The new tax on high-sugar drinks…is a welcome step in the direction of tackling child obesity, and I hope that parents will take the message on board by encouraging children and teenagers to drink and eat more healthily’.*

*Chris Evans (Labour Party/Co-op) (Islwyn)*

*Diabetes Related Complications 2016-06-07*

##### More needs to be done

Although pro-SDIL views were more frequently expressed than anti-SDIL views, there was a universal consensus from both those speaking in support of, neutral towards and against, the SDIL that more needs to be done by government to address childhood obesity.

*‘Although I have said that I welcome the creation of a soft drinks levy, in isolation it cannot address the levels of obesity that we see. I am disappointed that further restrictions on junk food, as recommended by the Health Committee, have not been developed further….Banning those adverts would make a big difference’.*

*Carol Monaghan (Scottish National Party) (Glasgow North West)*

*Soft Drinks Industry Levy: Funding for Sport in Schools 2017-01-10*

*‘…the Government had to be led kicking and screaming into agreeing some kind of sugar tax to reduce child obesity. The measures announced lack ambition. They should be broader and introduced sooner, but at least they are something. But where is the long-promised child obesity strategy?’*

*Baroness Joan Walmsley (Liberal Democrat) (West Derby)*

*Queen’s Speech 2016-05-19*

Suggestions of where these ‘next steps’ should be taken focused on the food environment and advertising and promotion and also on extending the SDIL to other product categories (in particular milk-based drinks), as well as food such as confectionary and sugary cereals.

*‘I also urge the Government to extend the sugary drinks levy to other drinks, including those in which sugar is added to milky products’.*

*Sarah Wollaston, Chair of Health Select Committee (Conservative Party) (Totnes)*

*Reducing Health Inequalities 2016-11-24*

*‘I look forward to the childhood obesity strategy that the Government are due to publish in the summer… A levy on sugar, or a sugar tax, is just one of the proposals that we [Health Select Committee] put forward, and just one of the things that needs to be done to tackle the problem of sugar consumption and obesity’.*

*Helen Whatley (Conservative Party) (Faversham & Mid Kent)*

*Budget Resolutions and Economic Situation 2016-03-17*

### Royal assent to implementation: April 2017 to April 2018

Between Royal Assent and implementation, there was a considerable reduction in instances where the SDIL was discussed, resulting in just one further theme.

#### Theme 3: the SDIL worked – what next?

Following royal assent, discussion about the SDIL predominately suggested the policy had already worked, mainly due to the early reformulation efforts by industry. However, although the frequency of debate on the SDIL reduced the arguments discussed previously that ‘more is needed’ persisted.

*‘It seems that the threat of the tax and the Government’s legislation on the soft drinks industry levy, due for implementation in April 2018, has already altered behaviour and the food industry is reformulating its products and reducing the sugar content’.*

*Baron Bernard Ribeiro (Conservative Party) (Ovington)*

*Queen’s Speech 2017-06-29*

##### SDIL as a template for future policy

Discussion described the SDIL as a template and proposed future policy suggestions based on its success, similar to the previous time period. This discussion worked to emphasise that the SDIL was a ground-breaking and novel policy.

*‘If we have established the principle with sugary drinks, there is no reason why we should not extend that approach to other foods, so that it will lead in the main part to reformulation’*

*Andrew Selous (Conservative Party) (South West Bedfordshire)*

*Junk Food Advertising and Childhood Obesity 2018-01-16*

##### SDIL revenue projections revised down

The discussion regarding hypothecated taxation persisted as SDIL revenue projections were revised downwards again and the government promised to continue to fill the shortfall.

*‘It was suggested in the draft proposals that the levy would raise an ambitious £520 million. However, the Chancellor announced…that its estimated revenue had been revised down to £380 million, and the Office for Budget Responsibility forecast in December, on the basis of the Government’s Red Book for the autumn Budget, that it would raise only £300 million. That is a whopping £220 million less than the Government’s original forecast, and a further £80 million less than the revised figure that the Chancellor provided in the spring Budget’.*

*Peter Dowd, Shadow Chief Secretary to the Treasury (Labour Party) (Bootle)*

*Finance (No. 2) Bill (Fourth Sitting) 2018-01-11*

A new argument was found, related to what may be next for future policy and the SDIL. Some expressed it was contradictory to introduce the SDIL to improve health, but at the same time cut budgets to services related to health.

*‘How can the sugar levy make sense when many other measures that might help to reduce childhood obesity are being cut? That is something that we will continuously raise when it comes to this levy’.*

*Clive Lewis, Shadow Secretary of State for Business, Energy and Industrial Strategy (Labour Party) (Norwich South)*

*Draft Soft Drinks Industry Levy (Enforcement) Regulations 2018-02-07*

### Post-implementation: April 2018 to March 2020

#### Theme 4: moving on from the SDIL

##### The SDIL is old news

After implementation, the argument that the SDIL ‘worked’ persisted; however, in this time period discussion shifted to it being ‘old news’. References to the SDIL were shorter and often used to support debate and arguments for the introduction of new policies. The arguments – that the SDIL is just one piece of the puzzle and that multiple strategies are needed – persisted and were the strongest theme throughout all time periods.

*‘I acknowledge that we have seen some helpful steps forward in recent years, such as the introduction of a sugar tax… but these are piecemeal and un-coordinated’.*

*Luciana Berger, Shadow Minister for Mental Health (Labour Party/Co-op) (Liverpool, Wavertree)*

*Health Impacts (Public Sector Duty) 2018-04-25*

*‘Will the Secretary of State confirm whether the Government’s second childhood obesity plan…will include meaningful policies such as restricting junk food advertising and the sale of energy drinks to children?*

*Sharon Hodgson, Shadow Minister for Public Health (Labour Party) (Washington & Sunderland West)*

*I agree with the honourable Lady that we need to do more, because this is a very serious issue. I think that she is being slightly unfair on our first initiative. The sugary drinks tax has been responsible for 45 million kg of sugar being removed from the market, which is enormously important for children. There is more to be done and I hope that we will be able to announce plans soon’.*

*Jeremy Hunt, The Secretary of State for Health and Social Care (Conservative Party) (South West Surrey)*

*Obesity 2018-05-08*

Post-implementation, the debate also included using the SDIL as an example or template for how future policies could work, including extending the SDIL to other drinks (milk-based and alcohol) as well as other food categories.

*‘The amount of sugar in soft drinks has been reduced by 11% in response to the industry levy… The Minister says that progress is not good enough, so why does he not introduce a levy on high-sugar food as well as the one on sugary drinks? Manufacturers would then reformulate the food that they produce’.*

*Diana Johnson (Labour Party) (Kingston upon Hull North)*

*Childhood Obesity 2018-06-19*

*‘The sugar tax is actually quite popular; I think any popular tax is a jolly good thing. I invite the Minister to initiate a few cross-party discussions on the extent to which sugar-laden goods and highly processed goods can be further taxed. If we can raise money for the NHS by taxing things – and being popular with it – I suggest that is a good thing’.*

*Lord Richard Balfe (Conservative Party) (Dulwich)*

*Obesity 2018-07-18*

Evaluating the success of the SDIL during this period was discussed only in relation to extending the SDIL to milk-based drinks, as a review of the exclusion of these had been promised in 2020.

*‘…When we announced the policy, we said that we would consider milk-based sugary drinks in 2020, which is when more information, including Public Health England data, will be available to inform that decision’.*

*Robert Jenrick, Exchequer Secretary to the Treasury (Conservative Party) (Newark)*

*Finance (no. 3) Bill (Eighth Sitting) 2018-12-06*

##### The SDIL is evidence of government taking action

As discussed in theme 3, the SDIL was discussed as world-leading and ground-breaking. In this time period, it was often used in discussions unrelated to the SDIL as a tool to demonstrate that the government is acting on public health. In particular, using the levy funds for breakfast clubs and school physical activity was seen as evidence of this.

*‘[in response to a question about restricting junk food marketing] We already have plans to tackle childhood obesity that are world leading. No other developed country has done anything as ambitious. Our soft drinks industry levy is a bold action that we are taking, and our sugar reduction programme will cut the amounts of sugar consumed by young people’.*

*Theresa May, The Prime Minister (Conservative Party) (Maidenhead)*

*Engagements 2018-04-25*

Discussion about the SDIL was sometimes used to redirect or equivocate. In particular, MP said that the SDIL helped demonstrate the government’s action in response to an unrelated question about the absence of action in other areas.

*‘I am grateful that the shadow Secretary of State has drawn attention to public health [in a question about health visitors] because the Government have been making significant progress in that area… we are addressing child obesity with the sugar tax, which is among a number of measures that the Government have been bringing forward’.*

*Steve Barclay, Minister of State for Health (Conservative Party) (North East Cambridgeshire)*

*NHS Workforce 2018-06-19*

##### Threats to the SDIL

Two distinct threats to the SDIL were present: concerns about litigation against the government based on the experiences of this in Mexico and the USA and explicit threats to reverse the SDIL made by Boris Johnson during his leadership campaign and subsequently when he took office.

*‘The Sports Minister has a responsible role to play in tackling obesity, so will she today publicly commit to resisting any call to scrap the sugar tax, even from her favoured candidate for Prime Minister?’*

*Rosena Allin-Khan, Shadow Minister for Sport (Labour Party) (Tooting)*

*Topical Questions 2019-07-04*

Government ministers responded to questions about this threat to repeal by discussing the success of the SDIL. They did not however directly respond that it would not be repealed in future.

*‘We can see how successful the soft drinks industry levy has been in how it has helped to reformulate sugary drinks, the amount of money it has raised that has been recycled into school sports, and the fact that it is changing people’s tastes and behaviour. The prevention Green Paper is in train; let us hope that he is pleased with what is announced in it’.*

*Seema Kennedy (Conservative Party) (South Ribble) Parliamentary Under-Secretary of State for Health and Social Care*

*NHS Dentists Cumbria 2019-07-03*

## Discussion

### Summary of main findings

This is the first study to examine parliamentary reaction to the UK SDIL and aims to explore how policies persist in the face of threats. Our analysis of debate transcripts demonstrates that Parliament mostly welcomed the SDIL when it was announced, but believed that more policy intervention was needed to address childhood obesity. Once the SDIL achieved royal assent and was made law, Parliament moved on from the policy and, even prior to implementation, noted that it had successfully achieved sugar reformulation in UK SSB. Following the implementation of the SDIL, Parliament moved on further, discussing the policy as a template for future policy intervention. These findings suggest that the mostly positive debate content surrounding the SDIL, particularly descriptions of it as a success prior to and after implementation, helped it withstand threats to repeal.

### Strengths and limitations

The use of Applied Thematic Analysis is a strength as it uses techniques to limit the influence of bias and subjectivity in descriptive analyses, for example multiple coders and detailed codebooks^(26)^. Applied Thematic Analysis thus generated a robust, descriptive account of parliamentary debate following the announcement of the SDIL. Although ‘true’ generalisability and replicability of the findings may not ever be achieved in qualitative research, using Applied Thematic Analysis to analyse this data means the work aligns more closely with post-positivist societal leanings to seek this. By selecting a method more acceptable to the traditional evidence-based paradigm of policy development, the findings of this work are more likely to be used to inform future policy decisions by those who prize post-positivism (e.g. biomedical statistical approaches) over interpretivism (e.g. qualitative approaches which integrate researcher interpretation throughout the analysis).

Thematic coding by hand of such a large dataset presented a challenge during the familiarisation period of analysis. Some coding techniques lend themselves to simple quantitative analyses; however, these were too labour-intensive to perform with the quantity of data included in this study. Therefore, the third-level quantitative analyses only informed the refinement of themes derived during second-level analysis and were not performed as independent quantitative analyses. The time-intensive hand-coding of a large data corpus also led to some analytic areas not being addressed, such as a breakdown of sentiment and themes by political party.

### Relationship to prior knowledge

Food industry arguments suggest there is insufficient good-quality evidence to demonstrate that SSB taxes work^(30)^. The food industry often argues for a high standard and volume of evidence prior to the commencement of regulation for health purposes, whereas our analysis shows that parliamentarians seem to make decisions based on more limited evidence supporting success. Research evaluating the SDIL confirms that reformulation of sugar out of SSB has occurred; by February 2019, 33.8% fewer drinks contained enough sugar to meet the minimum SDIL threshold^(19)^. Therefore, these assertions in Parliament, although premature, do appear to have been correct in suggesting that the SDIL successfully achieved its primary goals.

Complexity arguments surrounding obesity, e.g. that one policy cannot solve it, were found to be the most prevalent code during this analysis. Research exploring the use of these arguments in policymaking has previously found that they serve two purposes: to show health problems as so complex that they cannot be solved by policy change and to deflect responsibility towards individuals while obscuring government and industry inaction^(31)^. Further research has also found that industry documents often use the ‘impossible complexity of public health problems’ argument to deflect blame away from industry and onto individuals^(30)^. The discovery of these arguments, commonly used by the food and drink industry, within our findings could indicate that parliamentarians could either be influenced by the food industry (directly or indirectly) in their debate or these complexity arguments are simply pervasive in society.

### Interpretation and implications for policy and practice

After the implementation of the SDIL, threats to reverse it were made by both Boris Johnson and Liz Truss during their leadership campaigns and appointments as Prime Minister. This demonstrates the political uncertainty regarding more controversial policies like the SDIL, which future leaders may view differently from their political predecessors, even within the same political party (in this case, the Conservative Party). Our findings help to understand some of the resilience of the SDIL to these threats. Assertions that the SDIL had ‘worked’ were made as early as November 2016, 8 months following the announcement. These assertions also persisted and the narrative within parliamentary debate became that the SDIL was a successful policy for reducing sugar consumption. A continuing narrative of policy ‘success’ may have made the SDIL more resilient to attempts to repeal it, suggesting a strategy of making early claims of success (in the case of the SDIL related to reformulation) may be a useful tool in combatting opposition to announced public health policy.

Important external events occurring during the time these debates took place provide context to our findings. After the announcement in March 2016, the EU referendum was held. The decrease in parliamentary discussion following the SDIL royal assent in April 2017 could be due to this unexpected seismic political event, shifting parliamentary priorities from public health to Brexit. In addition, Prime Minister David Cameron and Chancellor George Osborne were responsible for the announcement and initial design of the SDIL. The result of the Brexit referendum, that the UK would leave the European Union, resulted in David Cameron’s resignation as Prime Minister and George Osborne’s resignation as Chancellor. Once Theresa May became Prime Minister, her political objectives may not have aligned in the same way to the SDIL^(32)^. Importantly, the decrease in SDIL discussion described thematically as parliamentarians ‘moving on from the SDIL’ could also represent the change in government and that the new Government’s policy priorities are no longer aligned with the SDIL.

Importantly, our analysis is limited to transcripts available from formal sessions of Parliament that follow strict rules and protocols. Parliamentary debate, however, is only the theatre in which politics is performed publicly. While it provides us an insight into reaction to the SDIL, the ‘true’ reaction and opinion about it, as well as some political decision-making, may have been expressed or conducted behind closed doors (e.g. in the Cabinet) or through other avenues. Therefore, interpretation of these findings should be made in the knowledge that these were expressed via a public platform.

### Future research

Building on our findings, future research could explore in more detail the debate transcripts prior to the announcement of the SDIL. Initial familiarisation was conducted on 2014–2016 SDIL-related debate; however, the debate content in this time period appeared to answer a different aim than that for the current study – namely, how did the SDIL come about? Although assessing parliamentary reaction to a policy helps provide a window to the policy process, how sugar taxation was debated prior to its announcement could compliment these insights. By exploring how SSB taxation was framed by parliamentarians, and how evidence was used to support arguments both for and against, insight into the development and ultimate adoption of an SSB levy in the UK could be gained. This would compliment previous work exploring how national newspapers portrayed the SDIL publicly^(33)^, and how industry reaction to it was communicated through the news media^(34)^.

However, parliamentary debate as described previously is a public platform. Therefore, a further complement to this work would be to explore behind-the-scenes political decision-making regarding the SDIL to explore what ‘really’ occurred prior to its announcement. Combined this work could help guide advocates in which approaches to encouraging greater regulation of the food and drink industry could translate to tangible changes in policy. Analyses could explore links between parliamentarians and industry, for example whether food and drink industry are located in their constituencies or if they have previously held jobs within these companies. Further, corroboration between statements made in debate could be interrogated using the Register of Members’ Financial Interests^(35)^ or statements made via social media. Finally, analyses could extend this work by comparing parliamentary debate on SSB taxes or public health policies internationally. Exploring whether similar patterns to this work are found elsewhere could indicate possible strategies to ensure the resilience of public health policy for example by emphasising its success as early as possible.

### Conclusion

Our work explored the UK parliamentary debate on the SDIL and found the policy had cross-party support, was deemed a ‘success’ prior to implementation and was often discussed as a good first step but that more needed to be done to combat obesity. Indications are that support was bolstered by the ring-fencing of funds for ‘good’ causes, the high profile of childhood obesity as a campaigning issue by celebrities and the scale of health problems associated with excess sugar consumption. Debate lessened on the SDIL after it became law through Royal Assent and further reduced following implementation. Developing politically broad-based parliamentary support for seemingly contentious public health policies and establishing an early narrative of policy success may help build policy resilience.

## Data Statement

The lead author has full access to the data reported in the manuscript.
